# Gene Network Analysis of Candidate Loci for Human Anorectal Malformations

**DOI:** 10.1371/journal.pone.0069142

**Published:** 2013-08-01

**Authors:** Emily H. M. Wong, Chun-Laam Ng, Vincent Chi-Hang Lui, Man-ting So, Stacey S. Cherny, Pak-Chung Sham, Paul Kwong-Hang Tam, Maria-Mercè Garcia-Barceló

**Affiliations:** 1 Department of Psychiatry, Li Ka Shing Faculty of Medicine, The University of Hong Kong, Hong Kong SAR, China; 2 Department of Surgery, Li Ka Shing Faculty of Medicine, The University of Hong Kong, Hong Kong SAR, China; 3 Center for Genomic Sciences, Li Ka Shing Faculty of Medicine, The University of Hong Kong, Hong Kong SAR, China; 4 Centre for Reproduction, Development, and Growth, Li Ka Shing Faculty of Medicine, The University of Hong Kong, Hong Kong SAR, China; 5 State Key Laboratory of Brain and Cognitive Sciences, The University of Hong Kong, Hong Kong SAR, China; Institut Jacques Monod, France

## Abstract

Anorectal malformations (ARMs) are birth defects that require surgery and carry significant chronic morbidity. Our earlier genome-wide copy number variation (CNV) study had provided a wealth of candidate loci. To find out whether these candidate loci are related to important developmental pathways, we have performed an extensive literature search coupled with the currently available bioinformatics tools. This has allowed us to assign both genic and non-genic CNVs to interrelated pathways known to govern the development of the anorectal region. We have linked 11 candidate genes to the WNT signalling pathway and 17 genes to the cytoskeletal network. Interestingly, candidate genes with similar functions are disrupted by the same type of CNV. The gene network we discovered provides evidence that rare mutations in different interrelated genes may lead to similar phenotypes, accounting for genetic heterogeneity in ARMs. Classification of patients according to the affected pathway and lesion type should eventually improve the diagnosis and the identification of common genes/molecules as therapeutic targets.

## Introduction

Anorectal malformations (ARMs, congenital obstruction of the anal opening) are among the most common birth defects requiring surgical treatment (2–5/10,000 live-births) and carry significant chronic morbidity. There is a wide spectrum of defects that differ in terms of malformation severity (ranging from anal stenosis to persistent cloaca) and the presence of different associated congenital anomalies. As shown in the genome-wide copy number variation (CNV) study we published recently [1], rare CNVs (either genic or non-genic) private to individual patients are likely to underlie the phenotype. Notably, the CNVs identified differ among patients indicating that ARMs are genetically heterogeneous diseases. Indeed, given the complexity of molecular events that take place during embryonic stages, impairments in any gene members of the pathways controlling the development of the anorectal region could lead to ARMs (or even lethality), thus accounting for the genetic heterogeneity observed among patients. Relevant pathways reported so far in animal studies include WNT, BMP and SHH.

To find out whether the CNVs identified in our study could be assigned to the afore mentioned pathways, we performed an extensive literature search which, coupled with the currently available bioinformatics tools, have allowed us to assign both genic and non-genic CNVs to interrelated pathways known to govern the development of the anorectal region.

## Results and Discussion

A total of 79 genes distributed among 67 genic-CNV regions (1 recurrent, 66 singletons) and 56 non-genic regions (1 recurrent, 55 singletons) were found uniquely disrupted in 88 out of 170 ARM patients analysed (Table S1 in File S1). These CNVs were not reported in the 11,943 healthy individuals from the Database of Genomic Variants (DGV), nor were they found in our 868 control individuals.

**Figure 1:**
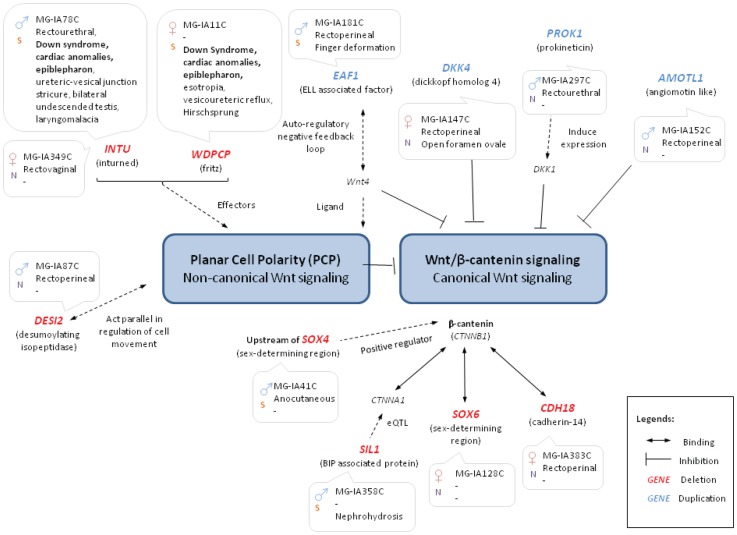
Candidate genes related to WNT signalling pathway. The relevance of the 11 candidate genes (in red: deleted; in blue: duplicated) to WNT signalling pathway is displayed. Sample ID and phenotypes of the patients with gene disruptions are also listed. N: Northern Chinese; S: Southern Chinese.

**Figure 2:**
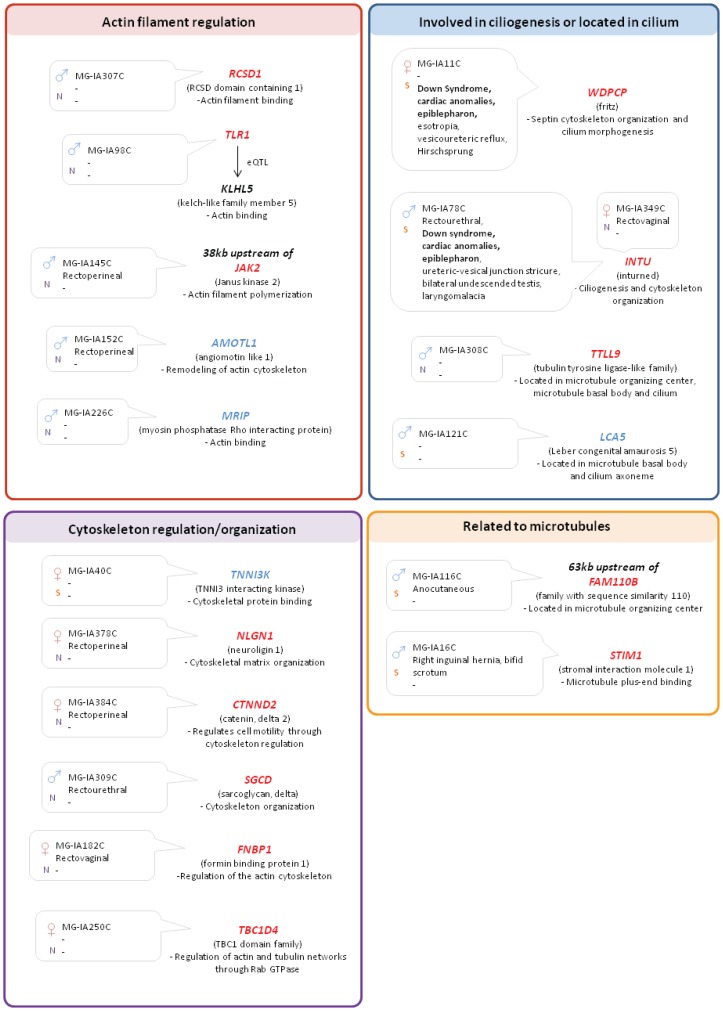
Candidate genes related to the cytoskeleton network. The relevance of the 17 candidate genes (in red: deleted; in blue: duplicated) to cytoskeleton network is displayed. Sample ID and phenotypes of the patients with gene disruptions are also listed. N: Northern Chinese; S: Southern Chinese.

Non-genic regions were scrutinized for the presence of regulatory elements controlling the expression of gene members of the relevant pathways. To this end, genomic regions were submitted to RegulomeDB [2], which detects regulatory signatures within the regions, including transcription factor binding sites (TFBS) and expression quantitative trait loci (eQTL) that can be linked to the expression of their target genes. Importantly, RegulomeDB summarizes the data recently released by ENCODE (the Encyclopedia of DNA Elements) [3]. The 79 genes uniquely disrupted by CNVs in patients, together with the TFBS and eQTL target genes, are denoted as “candidate genes” here. Details on the functions and characteristics of these genes were obtained from DAVID bioinformatics resources [4], NCBI Gene (http://www.ncbi.nlm.nih.gov/gene) and literature search. For those patients with multiple candidate genes disrupted, the most biologically plausible genes were selected and examined. The relationship among the genes or regulatory regions identified, together with the phenotypic characteristics of the patients are depicted in Figures 1 and 2.

Eleven candidate genes (each altered in a different patient) were assigned/linked to the WNT signalling pathway and one (*ESCIT*) to the WNT related BMP signalling pathway (see Table 1 and supplementary material in File S1 for details). Among those, the three duplicated genes (*DKK4, AMOTL*1 and *PROK1*) known to be antagonists of the WNT/β-catenin signalling [5]–[7] are essential for the development of the anorectal region (Table S2 in File S1). Importantly, excess of *DKK4* has been associated with the ARMs phenotype in mice [1]. Six genes related to β-catenin are affected by CNVs in six ARM patients respectively and are described as follows. Deletions of genes encoding β-catenin-binding proteins (*SOX6, CDH18* and *CTNND2*) and regulatory regions affecting the expression of genes encoding other β-catenin-binding proteins (target genes: *CTNNA1, SOX4)* were detected. In addition, a gene affecting the expression of β-catenin negative regulator (*EAF1)* is duplicated. Genes encoding effectors (*INTU* and *WDPCP*) of the planar cell polarity pathway (PCP; non-canonical WNT signalling) were deleted as well. Apart from PCP effectors, a deletion was observed in *DESI2*, which acts in parallel with the non-canonical WNT signalling pathway regulating cell movement during embryonic development. Planar cell polarity and WNT/β-catenin signalling are inter-related and they interact with other important signalling pathways during embryonic development, including the BMP signalling pathway. *ESCIT*, a cofactor of Smad proteins which mediate BMP signalling, was duplicated in an ARM patient. Defects in the above pathways are known to recapitulate the ARM phenotype in mice [8]–[10].

**Table 1:** List of 11 candidate genes related to the WNT signalling pathway together with one candidate gene (*ECSIT*) related to Bmp signalling pathway.

Gene	Chr	Start position	End position	Patient ID	Sex	Type of fistula	Associated Anomaly	Type^a^	Function
*PROK1*	1	111003201	111040235	MG-IA297C	M	Rectourethral	*N.D.*	DUP	Induces expression of *DKK1*, an antagonist of WNT signalling
*DESI2*	1	240986292	249219320	MG-IA87C	M	Rectoperineal	*N.D.*	DEL	Acts in parallel with non-canonical WNT signalling pathway to regulate cell movement
*WDPCP*	2	63515568	63564683	MG-IA11C	F	*N.D.*	Down Syndrome, cardiac anomalies, epiblepharon, esotropia, vesicoureteric reflux, Hirschsprung	DEL	Effector of Planar Cell Polarity
*EAF1*	3	15484269	15565683	MG-IA181C	M	Rectoperineal	Finger deformation	DUP	Regulates *WNT4* expression with EAF2/U19
*INTU*	4	128586241	128620622	MG-IA78C	M	Rectourethral	Down syndrome, cardiac anomalies, ureterovesical junction stricture, epiblepharon, undescended testis, laryngomalacia	DEL	Effector of Planar Cell Polarity
				MG-IA349C	F	Rectovaginal	*N.D.*	DEL	
*CDH18*	5	19970226	19975567	MG-IA383C	F	Rectoperineal	*N.D.*	DEL	Binds to β-catenin
*SIL1*	5	138291405	138307306	MG-IA358C	M	*N.D.*	Nephrohydrosis	DEL	eQTL of *CTNNA,* binds to β-catenin
192kb upstream of *SOX4*	6	21357289	21402434	MG-IA41C	M	Anocutaneous	*N.D.*	DEL	Overlaps with a TFBS (MEF-2). *SOX4*, the nearest gene, induces β-catenin expression
*DKK4*	8	36727529	49890132	MG-IA147C	F	Rectoperineal	Open foramen ovale	DUP	Antagonist of WNT/β-catenin signalling
*SOX6*	11	16253569	16285515	MG-IA128C	F	*N.D.*	*N.D.*	DEL	Binds to β-catenin
*AMOTL1*	11	91123671	99694305	MG-IA152C	M	Rectoperineal	*N.D.*	DUP	Antagonist of WNT/β-catenin signalling
*ECSIT*	19	11584725	11651810	MG-IA230C	M	Rectoperineal	*N.D.*	DUP	Coeffector for Smad proteins in Bmp signalling

The functions of these genes were extracted from DAVID bioinformatics resources, NCBI Gene or literature. Positions listed are from UCSC version hg19. *N.D.*: not described in the clinical records. ^a^Type of CNV: DEL, deletion; DUP, duplication.

We also detected the disruption of 17 genes that are functionally related to or located in cytoskeleton (Table 2). Of note, the cytoskeletal network leading to ciliogenesis, intracellular trafficking and cell movements, is regulated by WNT signalling, and especially by PCP [11], [12].

**Table 2:** List of 17 candidate genes related to cytoskeleton.

Gene	Chr	Start position	End position	Patient ID	Sex	Type of fistula	Associated Anomaly	Type^a^	Relevance to cytoskeleton
*TNNI3K*	1	74699099	74712181	MG-IA40C	F	*N.D*.	*N.D.*	DUP	Cytoskeletal protein binding
*RCSD1*	1	167665064	167667620	MG-IA307C	M	*N.D*.	*N.D.*	DEL	Actin filament binding
*WDPCP*	2	63515568	63564683	MG-IA11C	F	*N.D.*	Down Syndrome, cardiac anomalies, epiblepharon, esotropia, vesicoureteric reflux, Hirschsprung	DEL	Involved in septin cytoskeleton organization and cilium morphogenesis
*NLGN1*	3	173462655	173494302	MG-IA378C	F	Rectoperineal	*N.D.*	DEL	Involved in cytoskeletal matrix organization at active zone
*TLR1*	4	38799710	38820986	MG-IA98C	M	*N.D.*	*N.D.*	DEL	eQTL of *KLHL5*, involved in actin binding
*INTU*	4	128586241	128620622	MG-IA78C	M	Rectourethral	Down syndrome, cardiac anomalies, ureterovesical junction stricture, epiblepharon, undescended testis, laryngomalacia	DEL	Controls ciliogenesis and cytoskeleton organization
				MG-IA349C	F	Rectovaginal	*N.D.*	DEL	
*CTNND2*	5	11335373	11337346	MG-IA384C	F	Rectoperineal	*N.D.*	DEL	Regulates cell motility through regulating cytoskeleton
*SGCD*	5	155367923	155371739	MG-IA309C	M	Rectourethral	*N.D*.	DEL	Involved in cytoskeleton organization
*LCA5*	6	80115863	80353483	MG-IA121C	M	*N.D*.	*N.D*.	DUP	Locates in microtubule basal body and cilium axoneme
63kb upstream of *FAM110B*	8	58841890	58844022	MG-IA116C	M	Anocutaneous	*N.D.*	DEL	*FAM110B* locates in microtubule organizing center
38kb upstream of *JAK2*	9	4921639	4947510	MG-IA145C	M	Rectoperineal	*N.D*.	DEL	*JAK2* involved in actin filament polymerization
*FNBP1*	9	132771351	132803206	MG-IA182C	F	Rectovaginal	*N.D.*	DEL	Effector of Rho, locates in cytoskeleton
*STIM1*	11	3951337	3970412	MG-IA16C	M	Rectovesical	Right indirect inguinal hernia, bifid scrotum	DEL	Involved in microtubule plus-end binding
*AMOTL1*	11	91123671	99694305	MG-IA152C	M	Rectoperineal	*N.D.*	DUP	Induces the remodeling of the actin cytoskeleton through its association with p80-angiomotin-containing complex
*TBC1D4*	13	75978839	75998520	MG-IA250C	F	*N.D.*	*N.D.*	DEL	Rab GTPase activator
*MPRIP*	17	16764978	17218859	MG-IA226C	M	*N.D.*	*N.D.*	DUP	Involved in actin binding
*TTLL9*	20	30533883	30552344	MG-IA308C	M	*N.D.*	*N.D.*	DEL	Locates in microtubule organizing center, microtubule basal body and cilium

The relevance of these genes to the cytoskeleton was extracted from DAVID bioinformatics resources, NCBI Gene or literature. Positions listed are from UCSC version hg19. *N.D.*: not described in the clinical records. ^a^Type of CNV: DEL, deletion; DUP, duplication.

Among the 17 genes, 5 are related to actin filament regulation or binding (*RCSD1, KLHL5, JAK2, AMOTL1*and *MRIP*), 4 are involved in ciliogenesis or located in the cilium (*INTU, WDPCP, LCA5* and *TTLL9*) and 2 are associated to microtubules (*FAM110B* and *STIM1*). Six other genes (*TNNI3K, NLGN1, CTNND2, SGCD, FNBP1* and *TBC1D4)* are involved in regulation and/or organization of the cytoskeleton network. Regulation of the cytoskeleton would probably be important for the downstream process of WNT signalling, though the implication remains unknown.

Close examination of our previous data has allowed us to link 27 candidate genes to interrelated pathways of relevance to the development of the anorectal region. Interestingly, we noted that for WNT signalling, candidate genes with similar function were disrupted by the same type of CNVs in ARM patients: (1) WNT signalling antagonists and β-catenin negative regulators were duplicated; (2) WNT signalling modulators (β-catenin binding proteins and PCP effectors) were all deleted. However, for those genes related to the cytoskeleton network, we did not observe any link between their function and type of lesion by which they were interrupted.

Some features that hint at the pathogenicity, including the mode of inheritance and functional impact of the duplication cannot, however, be established as parental samples are not available and the location of the duplicated copy in the genome is unknown.

Validating all these CNVs in the 27 regions using another technology poses a challenge given limited DNA availability. However, the chance of these CNVs being false positives is low as the CNVs are called by at least two programs. The rarity of the CNVs in these 27 regions, together with the functional regions intersected suggest that indeed these CNVs play a role in ARMs.

## Conclusions

The data presented here confirm that ARMs are genetically heterogeneous diseases. Also, the fact that genes with similar function within a network are disrupted by the same type of lesion may provide interesting insights into network interacting mechanisms. Ideally, classification of the patients according to type of genetic lesion and pathway affected should lead to improved clinical diagnosis, as well as to the identification of a putative common downstream gene or molecule which could be used as a target for future treatment development.

## Materials and Methods

### Subjects and ethics statement

The overall study was approved by the institutional review board of The University of Hong Kong together with the Hospital Authority (IRB: UW 07–321). Blood samples were drawn from all participants after obtaining written informed consent from patients or from parents, guardians or next of kin on behalf of minors.

#### ARM patients

170 Chinese sporadic ARM patients (isolated or with additional associated anomalies) had prospectively been collected throughout Hong Kong and Mainland China. All patients included in this study went through renal ultrasound, lumbosacral radiography and ECHO cardiography. Patients were defined as syndromic if associated anomalies were observed in addition to ARMs.

#### Controls

We included 868 individuals who are phenotypically normal from other studies as controls: 111 controls from a hypertension study [13] and 757 individuals from an osteoporosis study [14].

### Whole-genome CNV scan

The whole-genome scan was performed at deCODE Genetics (Reykjavik, Iceland) using Illumina Human 610-Quad BeadChips which assay 599,011 SNPs across the genome and 21,890 intensity-only monomorphic CNV probes. The B allele frequency (BAF) and log R ratio (LRR) were provided by deCODE for CNV calling. Details on CNV prediction and quality controls were described previously [1].

### Disease gene mapping

#### Gene-based analysis

CNV calls in ARM patients were compared against our controls (N = 868) and the 11,943 healthy individuals from the DGV. We selected those genes that were uniquely disrupted by CNVs in ARM patients, but not in controls nor healthy individuals in DGV, for further analysis. Details on the functions and characteristics of these genes were obtained from DAVID (the database for annotation, visualization and integrated discovery) bioinformatics resources [4], NCBI Gene (http://www.ncbi.nlm.nih.gov/gene) and literature search.

#### Patient-unique non-genic CNV regions

For those non-genic CNV regions that were uniquely disrupted in ARM patients, we checked if they overlapped with any transcription factor binding sites or expression quantitative trait loci based on the database RegulomeDB [2], which summarized data from ENCODE. Functions of the TFBS nearby genes and eQTL target genes were also obtained from DAVID bioinformatics resources, NCBI Gene and literature search.

#### Expression in mouse embryo

To carefully scrutinize the genes related to ARMs, the list of “candidate genes” has been checked against Eurexpress (Mouse Gene Expression Atlas) for their expression of candidate genes in mouse embryo. Genes have been then categorized into four groups: (1) “with regional signal” if localized expression is observed in the urorectal organ, (2) “without regional signal” if the gene is expressed in the urorectal organ but is not specifically localized in a region, (3) “no signal” if it is not expressed at all in the urorectal organ, and (4) “not available in database” if there is no information on the expression of that gene. Those candidate loci that are not expressed in the mouse urorectal organ (i.e. category 3) have been excluded from the analysis.

## Supporting Information

File S1 Supporting text and tables. **Table S1:** List of the 79 genes and 56 non-genic regions uniquely disrupted in ARMpatients but neither in our 868 controls nor in 11,943 healthy individuals from DGV. **Table S2:** Gene expression during development of the mouse anorectal region (from Eurexpress; Mouse Gene Expression Altas).
(DOC)
