# Supplementary material for: Gene Network Analysis of Candidate Loci for Human Anorectal Malformations
Source: PLoS One. 2013 Aug 1;8(8):e69142. doi: 10.1371/journal.pone.0069142 (PMC3731316; doi:10.1371/journal.pone.0069142)
Supplement: File S1 — Table S1: List of the 79 genes and 56 non-genic regions uniquely disrupted in ARMpatients but neither in our 868 controls nor in 11,943 healthy individuals from DGV. Table S2: Gene expression during development of the mouse anorectal region (from Eurexpress; Mouse Gene Expression Altas). (DOC) [file pone.0069142.s001.doc]

# Gene network analysis of candidate loci for human anorectal malformations

# Supplementary materials and methods

# RESULTS

**Relevance of the candidate genes to WNT signaling pathway**

1. *DKK4*

*DKK4* encodes a secreted protein member of dickkopf (*DKK*) family of *WNT* regulators. *DKK*s, together with *WNT* secreted proteins play an important role in antero-posterior axial patterning, limb development, somitogenesis and eye formation. During development, *DKK4* competes with *WNT* ligands for the co-receptors, thus antagonizing *WNT* signaling pathway.

1. *AMOTL1*

*AMOTL1* regulates embryonic development by trapping β-catenin in the Rab11-positive recycling endosomes, reducing level of β-catenin in the cytosol and nucleus, thus inhibiting WNT/β-catenin signaling.

1. *PROK1*

*PROK1* inhibits WNT/β-catenin signaling by inducing the expression of *DKK1*, which binds to low density lipoprotein receptor-related protein-6 (LRP6), the co-receptor of WNT/β-catenin signaling, and prevents the Frizzled-WNT-LRP6 complex formation in response to WNTs.

1. *SOX6*

Encoding β-catenin-binding protein

1. *CDH18*

Encoding β-catenin-binding protein

1. *CTNND2*

Encoding β-catenin-binding protein

1. *CTNNA1* (target gene)

The eQTL site of *CTNNA1* (which encodes β-catenin-binding protein) in the intronic region of gene *SIL1* was deleted in one of the ARMs patients.

1. *SOX4* (target gene)

Deletion of the binding site of MEF-2, an important transcription factor for embryonic development, on chromosome 6p22.3 was observed in an ARMs patient. This might affect the expression of the nearest gene, *SOX4* which induces β-catenin expression.

1. *EAF1*

*EAF1*, which negatively regulates *WNT4* through its interaction with EAF2/U19, was duplicated in an ARMs patient. *WNT4* regulates the subcellular localization of β-catenin by redirecting it to the cell membrane, thus deregulation of *WNT 4* would also affect WNT/β-catenin signaling.

1. *INTU*

*INTU*, encoding effectors of planar cell polarity (PCP, a non-canonical WNT signaling), was deleted in two ARMs patients. Disruption in *INTU* would impair planar cell polarity and ciliogenesis, thus affect hedgehog signaling subsequently.

1. *WDPCP*

*WDPCP*, encoding effectors of planar cell polarity (PCP, a non-canonical WNT signaling), was deleted in an ARMs patient. Defects in *WDPCP*, which controls the localization of the cytoskeleton protein septin, would affect planar cell polarity, cell movement and ciliogenesis.

1. *ESCIT*

*ESCIT*, a cofactor of Smad proteins which mediate BMP signaling, was duplicated in an ARM patient. Defect in BMP signaling has been shown to affect cloaca development, and display the phenotype of ARMs. BMP signaling interacts with WNT signaling during embryonic development.

| **Table S1**: List of the 79 genes and 56 non-genic regions uniquely disrupted in ARMpatients but neither in our 868 controls nor in 11,943 healthy individuals from DGV. | | | | | | | | | |
| --- | --- | --- | --- | --- | --- | --- | --- | --- | --- |
| Chr | Start position | End position | Patient ID | Sex | Type of fistula | Associated Anomaly | Length (in kb) | CNV type | Gene |
| 2 | 221826031 | 221862562 | MG-B304 | M | Rectourethral | *N.D.* | 36.531 | DUP |  |
| 1 | 67993077 | 67998989 | MG-IA100C | F | Rectoperineal | *N.D.* | 5.912 | DEL |  |
| 1 | 186652395 | 186682875 |  |  |  |  | 30.48 | DEL | *PTGS2* |
| 17 | 70919212 | 70948535 |  |  |  |  | 29.323 | DEL | *SLC39A11* |
| 6 | 153529163 | 153688995 | MG-IA105C | M | Rectovesical | Bifid scrotum | 159.84 | DUP | *RPL27AP6* |
| 8 | 92364100 | 92398290 |  |  |  |  | 34.19 | DEL | *SLC26A7* |
| 7 | 48329838 | 48336844 | MG-IA110C | M | Rectoperineal | *N.D.* | 7.006 | DEL | *ABCA13* |
| 8 | 58841890 | 58844022 | MG-IA116C | M | Anocutaneous | *N.D.* | 2.132 | DEL |  |
| 2 | 63515568 | 63564683 | MG-IA11C | F | *N.D.* | Down Syndrome, cardiac anomalies, epiblepharon, esotropia, vesicoureteric reflux, Hirschsprung | 49.115 | DEL | *WDPCP* |
| 8 | 78440688 | 78478497 |  |  |  |  | 37.809 | DEL |  |
| 2 | 68109558 | 68250296 | MG-IA121C | M | *N.D.* | *N.D.* | 140.738 | DEL |  |
| 6 | 80115863 | 80353483 |  |  |  |  | 237.62 | DUP | *LCA5* |
| 4 | 131277890 | 131340652 | MG-IA122C | M | *N.D.* | *N.D.* | 62.762 | DEL |  |
| 5 | 67405115 | 67409074 |  |  |  |  | 3.959 | DEL |  |
| 5 | 92024632 | 92040221 |  |  |  |  | 15.589 | DEL |  |
| 12 | 70727924 | 70768681 |  |  |  |  | 40.757 | DEL | *CNOT2, KCNMB4* |
| 7 | 3898418 | 3915564 | MG-IA124C | M | Rectourethral | Prostatic utricle | 17.146 | DEL | *SDK1* |
| 10 | 36992914 | 37096929 |  |  |  |  | 104.015 | DUP |  |
| 19 | 15989040 | 16012612 |  |  |  |  | 23.572 | DEL | *CYP4F2* |
| 1 | 103354138 | 103380128 | MG-IA128C | F | *N.D.* | *N.D.* | 25.99 | DEL | *COL11A1* |
| 4 | 65556925 | 65579130 |  |  |  |  | 15.237 | DEL |  |
| 11 | 16253569 | 16285515 |  |  |  |  | 31.946 | DEL | *SOX6* |
| 13 | 87140651 | 87159109 |  |  |  |  | 18.458 | DEL |  |
| 8 | 93810859 | 93837436 | MG-IA135C | M | *N.D.* | *N.D.* | 26.577 | DEL |  |
| 6 | 56917538 | 56929863 | MG-IA138C | F | *N.D.* | *N.D.* | 12.325 | DEL | *KIAA1586* |
| 9 | 4921639 | 4947510 | MG-IA145C | M | Rectoperineal | *N.D.* | 25.871 | DEL |  |
| 5 | 50622582 | 51704435 | MG-IA147C | F | Rectoperineal | Open foramen ovale | 1081.86 | DUP | *ISL1* |
| 8 | 36727529 | 49890132 |  |  |  |  | 13206 | DUP | *ZNF703, ERLIN2, PROSC, BRF2, RAB11FIP1, GOT1L1, ADRB3, ASH2L, STAR, DDHD2, PPAPDC1B, FGFR1, TACC1, C8orf4, GOLGA7, GINS4, AGPAT6, NKX6-3, AP3M2, DKK4, C8orf40, CHRNA6, THAP1, CEBPD, SNAI2* |
| 15 | 98373118 | 98377630 |  |  |  |  | 4.512 | DEL | *LOC91948* |
| 11 | 91123671 | 99694305 | MG-IA152C | M | Rectoperineal | *N.D.* | 8436.2 | DUP | *TAF1D, C11orf54, MED17, FOLR4, GPR83, PIWIL4, AMOTL1, CWC15, KDM4D, SFRS2B, ENDOD1, FAM76B, MTMR2, CCDC82, JRKL* |
| 2 | 228644789 | 228652593 | MG-IA161C | M | Rectoperineal | *N.D.* | 7.804 | DEL |  |
| 20 | 57913395 | 57934450 | MG-IA162C | F | Rectovaginal | *N.D.* | 21.055 | DUP |  |
| 22 | 18822486 | 21465780 |  |  |  |  | 2593.3 | DEL | *SERPIND1, SNAP29* |
| 11 | 3951337 | 3970412 | MG-IA16C | M | Rectovesical | Right inguinal hernia, bifid scrotum | 19.075 | DEL | *STIM1* |
| 5 | 105039993 | 105079683 | MG-IA170C | M | *N.D.* | Down syndrome, cardiac anomalies, omphalocele | 39.69 | DEL |  |
| 16 | 87053729 | 87069983 | MG-IA179C | F | Rectovaginal | *N.D.* | 16.254 | DEL |  |
| 3 | 15484269 | 15565683 | MG-IA181C | M | Rectoperineal | Finger deformation | 81.414 | DUP | *EAF1, COLQ* |
| 6 | 150114745 | 150284435 |  |  |  |  | 169.69 | DUP | *RAET1G, ULBP2* |
| 9 | 132771351 | 132803206 | MG-IA182C | F | Rectovaginal | *N.D.* | 31.855 | DEL | *FNBP1* |
| 8 | 34185162 | 34197587 | MG-IA187C | F | Rectovaginal | Cardiac anomalies, microcephaly | 12.425 | DUP |  |
| 20 | 58326432 | 58544820 | MG-IA192C | M | *N.D.* | Cardiac anomaly | 218.39 | DUP | *SYCP2, PPP1R3D* |
| 1 | 235315543 | 237582758 | MG-IA195C | M | Anocutaneous | *N.D.* | 2267.22 | DEL | *RBM34, MTR* |
| 20 | 5647738 | 5659444 |  |  |  |  | 11.706 | DUP |  |
| 5 | 98941127 | 98967037 | MG-IA200C | F | Rectal navicular fossa | *N.D.* | 25.91 | DEL |  |
| 1 | 146501348 | 147841663 | MG-IA201C | F | Rectoperineal | *N.D.* | 1340.32 | DUP | *ACP6* |
| 15 | 74499407 | 74705682 | MG-IA204C | M | *N.D.* | Solitary kidney | 206.28 | DUP | *CCDC33* |
| 7 | 117138947 | 117150118 | MG-IA207C | F | Rectal navicular fossa | Cardiac anomalies | 11.171 | DEL | *CFTR* |
| 2 | 230030405 | 230034082 | MG-IA209C | M | Rectourethral | *N.D.* | 3.677 | DEL | *PID1* |
| 2 | 77103719 | 77113949 | MG-IA210C | M | Rectourethral | *N.D.* | 10.23 | DEL | *LRRTM4* |
| 2 | 78772214 | 78808678 |  |  |  |  | 36.464 | DEL |  |
| 2 | 155953969 | 155982775 |  |  |  |  | 28.806 | DEL |  |
| 2 | 191752327 | 191789907 |  |  |  |  | 37.58 | DEL | *GLS* |
| 5 | 44714330 | 44783255 |  |  |  |  | 68.925 | DEL |  |
| 5 | 129623638 | 129659729 |  |  |  |  | 36.091 | DEL |  |
| 6 | 102911869 | 103015795 |  |  |  |  | 103.926 | DEL |  |
| 11 | 24190190 | 24223001 |  |  |  |  | 32.811 | DEL |  |
| 1 | 64883923 | 64925891 | MG-IA216C | M | Rectourethral | *N.D.* | 41.968 | DEL |  |
| 17 | 16764978 | 17218859 | MG-IA226C | M | *N.D.* | *N.D.* | 453.89 | DUP | *TNFRSF13B, MPRIP, PLD6* |
| 1 | 159486328 | 159602473 | MG-IA228C | F | Rectal navicular fossa | *N.D.* | 116.145 | DEL | *OR10J5, APCS* |
| 4 | 39769586 | 39812247 |  |  |  |  | 42.661 | DEL | *UBE2K* |
| 11 | 24751895 | 24761831 | MG-IA229C | F | *N.D.* | *N.D.* | 9.936 | DUP | *LUZP2* |
| 19 | 11584725 | 11651810 | MG-IA230C | M | Rectoperineal | *N.D.* | 67.085 | DUP | *ELAVL3, ZNF653, ECSIT, CNN1* |
| 14 | 43471987 | 43530289 | MG-IA234C | M | *N.D.* | *N.D.* | 58.302 | DEL |  |
| 9 | 2515607 | 2531098 | MG-IA235C | M | *N.D.* | *N.D.* | 15.491 | DEL | *FLJ35024* |
| 20 | 19695350 | 19699148 |  |  |  |  | 3.798 | DEL | *SLC24A3* |
| 3 | 95731858 | 95752567 | MG-IA240C | F | *N.D.* | *N.D.* | 20.709 | DEL |  |
| 4 | 171813412 | 171842726 |  |  |  |  | 29.314 | DEL |  |
| 5 | 17126682 | 17137175 | MG-IA241C | M | *N.D.* | *N.D.* | 10.493 | DEL | *LOC285696* |
| 16 | 70662323 | 70677785 | MG-IA243C | F | *N.D.* | Down syndrome, cardiac anomalies | 15.462 | DEL | *IL34* |
| 4 | 65556925 | 65579130 | MG-IA245C | F | *N.D.* | *N.D.* | 22.205 | DEL |  |
| 17 | 21121136 | 21177799 | MG-IA249C | M | *N.D.* | *N.D.* | 56.664 | DUP | *TMEM11, C17orf103* |
| 13 | 75978839 | 75998520 | MG-IA250C | F | *N.D.* | *N.D.* | 19.681 | DEL | *TBC1D4* |
| 12 | 87534087 | 87636831 | MG-IA271C | M | Rectourethral | *N.D.* | 102.744 | DEL |  |
| 19 | 32126212 | 32139459 | MG-IA273C | F | Rectovesical | Kidney anomalies, omphalocele | 13.247 | DEL |  |
| 3 | 98611240 | 98674108 | MG-IA27C | M | *N.D.* | Kidney anomalies | 62.868 | DUP | *DCBLD2* |
| 3 | 164207439 | 164212378 | MG-IA280C | F | *N.D.* | *N.D.* | 4.939 | DEL |  |
| 1 | 184330372 | 184344022 | MG-IA281C | M | Rectourethral | *N.D.* | 13.65 | DEL |  |
| 5 | 164252520 | 164339317 | MG-IA282C | M | *N.D.* | *N.D.* | 86.797 | DEL |  |
| 15 | 55279251 | 55290575 | MG-IA286C | M | *N.D.* | Down syndrome | 11.324 | DEL |  |
| 16 | 4778609 | 4821698 | MG-IA294C | M | *N.D.* | *N.D.* | 43.089 | DUP | *ANKS3, C16orf71, ZNF500* |
| 5 | 151929836 | 151931892 | MG-IA295C | M | Rectourethral | *N.D.* | 2.056 | DEL |  |
| 1 | 111003201 | 111040235 | MG-IA297C | M | Rectourethral | *N.D.* | 37.04 | DUP | *PROK1* |
| 6 | 131499442 | 132103504 | MG-IA299C | F | *N.D.* | *N.D.* | 604.07 | DUP | *ARG1, MED23* |
| 9 | 135883563 | 135888516 | MG-IA301C | M | *N.D.* | *N.D.* | 4.953 | DEL |  |
| 16 | 86639662 | 86654474 | MG-IA303C | M | Rectourethral | *N.D.* | 14.812 | DEL |  |
| 1 | 167665064 | 167667620 | MG-IA307C | M | *N.D.* | *N.D.* | 2.556 | DEL | *RCSD1* |
| 8 | 97908518 | 98059046 |  |  |  |  | 150.528 | DEL | *PGCP* |
| 20 | 30533883 | 30552344 | MG-IA308C | M | *N.D.* | *N.D.* | 18.461 | DEL | *TTLL9, PDRG1, XKR7* |
| 5 | 155367923 | 155371739 | MG-IA309C | M | Rectourethral | *N.D.* | 3.816 | DEL | *SGCD* |
| 12 | 7477298 | 7890549 | MG-IA312C | M | Rectourethral | *N.D.* | 413.26 | DUP | *ACSM4, CD163* |
| 2 | 118873587 | 118879253 | MG-IA313C | M | Rectourethral | *N.D.* | 5.666 | DEL |  |
| 3 | 67336952 | 67422015 | MG-IA319C | F | *N.D.* | *N.D.* | 85.063 | DEL | *SUCLG2* |
| 6 | 137692864 | 137701907 |  |  |  |  | 9.043 | DEL |  |
| 5 | 116831336 | 116843143 | MG-IA323C | F | Vestibular fistula | *N.D.* | 11.807 | DEL | *LOC728342* |
| 3 | 37474032 | 37483387 | MG-IA334C | M | *N.D.* | *N.D.* | 9.355 | DEL | *C3orf35* |
| 4 | 172516584 | 172520744 | MG-IA338C | M | *N.D.* | Cardiac and kidney anomalies | 4.16 | DEL |  |
| 13 | 82516412 | 82518251 | MG-IA33C | M | Rectocutaneous | Obstructive sleep apnea syndrome | 1.839 | DEL |  |
| 14 | 38625943 | 38632531 | MG-IA342C | M | *N.D.* | *N.D.* | 6.588 | DEL |  |
| 4 | 128586241 | 128620622 | MG-IA349C | F | Rectovaginal | *N.D.* | 34.381 | DEL | *INTU* |
| 14 | 83454485 | 83463042 |  |  |  |  | 8.557 | DEL |  |
| 2 | 223674322 | 223693230 | MG-IA353C | F | *N.D.* | *N.D.* | 18.908 | DEL |  |
| 7 | 12941630 | 12953229 |  |  |  |  | 11.599 | DEL |  |
| 2 | 181659620 | 181679423 | MG-IA358C | M | *N.D.* | Nephrohydrosis | 19.803 | DUP |  |
| 5 | 138291405 | 138307306 |  |  |  |  | 15.901 | DEL | *SIL1* |
| 6 | 31807305 | 31816982 |  |  |  |  | 9.677 | DEL | *C6orf48* |
| 2 | 70796257 | 70815191 | MG-IA365C | M | *N.D.* | *N.D.* | 18.934 | DEL |  |
| 9 | 93433370 | 93464050 | MG-IA370C | F | Rectoperineal | *N.D.* | 30.68 | DEL |  |
| 3 | 173462655 | 173494302 | MG-IA378C | F | Rectoperineal | *N.D.* | 31.647 | DEL | *NLGN1* |
| 12 | 46709251 | 46720819 | MG-IA381C | F | Rectoperineal | *N.D.* | 11.568 | DUP |  |
| 3 | 153174119 | 153202348 | MG-IA382C | F | *N.D.* | *N.D.* | 28.229 | DEL | *C3orf79* |
| 15 | 96108853 | 96126414 |  |  |  |  | 17.561 | DEL |  |
| 5 | 19970226 | 19975567 | MG-IA383C | F | Rectoperineal | *N.D.* | 5.341 | DEL | *CDH18* |
| 5 | 11335373 | 11337346 | MG-IA384C | F | Rectoperineal | *N.D.* | 1.973 | DEL | *CTNND2* |
| 1 | 74157945 | 74405054 | MG-IA40C | F | *N.D.* | *N.D.* | 247.109 | DUP |  |
| 1 | 74699099 | 74712181 |  |  |  |  | 13.082 | DUP | *TNNI3K* |
| 6 | 21357289 | 21402434 | MG-IA41C | M | Anocutaneous | *N.D.* | 45.145 | DEL |  |
| 14 | 38259552 | 38318447 | MG-IA4C | M | Anocutaneous | *N.D.* | 58.895 | DEL | *TTC6* |
| 4 | 128586241 | 128620622 | MG-IA78C | M | Rectourethral | Down syndrome, cardiac anomalies, ureterovesical junction stricture, epiblepharon, undescended testis, laryngomalacia | 34.381 | DEL | *INTU* |
| 15 | 26993659 | 26997105 | MG-IA80C | M | Rectourethral | *N.D.* | 3.446 | DEL | *GABRB3* |
| 1 | 240986292 | 249219320 | MG-IA87C | M | Rectoperineal | *N.D.* | 8233.03 | DEL | *DESI2* |
| 1 | 19851016 | 19882170 | MG-IA93C | F | *N.D.* | Down syndrome, cardiac anomalies | 31.154 | DUP |  |
| 2 | 4981785 | 4988246 | MG-IA97C | M | *N.D.* | *N.D.* | 6.461 | DUP |  |
| 4 | 38799710 | 38820986 | MG-IA98C | M | *N.D.* | *N.D.* | 21.276 | DEL | *TLR1* |
| 10 | 18316940 | 18336330 |  |  |  |  | 19.39 | DUP | *SLC39A12* |
| 2 | 56538780 | 56638856 | MG-IA9C | F | *N.D.* | *N.D.* | 100.076 | DEL | *CCDC85A* |
| Positions as in UCSC version hg19. *N.D.*, not described in the clinical record. | | | | | | | | | |

| **Table S2:** Gene expression during development of the mouse anorectal region (from Eurexpress; Mouse Gene Expression Altas) | | |
| --- | --- | --- |
| **With Regional Signal** | | |
| **GENES** | **Region(s)** | **Signal Intensity (Strong/moderate/weak)** |
| *Dkk4* | Surface ectoderm of genital tubercle | moderate |
| *Amotl1* | Surface ectoderm of genital tubercle, Endoderm of urogenital sinus and apical genital mesenchyme | Strong |
| *Dcbld2* | Apical Genital tubercle mesenchyme, Bladder Mesenchyme | Strong |
| *Sox4* | Surface ectoderm and mesenchyme of Genital tubercle. Urorectal septum mesenchyme | Strong |
| *Fnbp1* | Genital tubercle mesenchyme | moderate |
| *Tgfa* | Surface ectoderm and urogenital sinus endoderm | Weak |
| *Ptgs2* | Surface ectoderm of genital tubercle | Weak |
| **Without Regional Signal** | | |
| **GENES** | **Region(s)** | **Signal Intensity (Strong/moderate/weak)** |
| *Med23* | Genital tubercle Mesenchyme (No Distinct Pattern) | Strong |
| *Eaf1* | Genital tubercle Mesenchyme (No Distinct Pattern) | Strong |
| *Ecsit* | Caudal Mesenchyme (No distinct Pattern) | moderate |
| *Sil1* | Caudal Mesenchyme (No distinct Pattern) | moderate |
| **No Signal** | | |
| *Nlgn1, Ctnnd2* | | |
| **Not available in the Database** | | |
| *Intu, Prok1, Wdpcp, Desi2, Mprip, Sox6, Cdh18, Dam110b* | | |

# REFERENCES

1. Niehrs C (2006) Function and biological roles of the Dickkopf family of Wnt modulators. Oncogene 25: 7469-7481.

2. Li Z, Wang Y, Zhang M, Xu P, Huang H, et al. (2012) The Amotl2 gene inhibits Wnt/beta-catenin signaling and regulates embryonic development in zebrafish. J Biol Chem 287: 13005-13015.

3. Macdonald LJ, Sales KJ, Grant V, Brown P, Jabbour HN, et al. (2011) Prokineticin 1 induces Dickkopf 1 expression and regulates cell proliferation and decidualization in the human endometrium. Mol Hum Reprod 17: 626-636.

4. Li Y, Lu W, King TD, Liu CC, Bijur GN, et al. (2010) Dkk1 stabilizes Wnt co-receptor LRP6: implication for Wnt ligand-induced LRP6 down-regulation. PLoS One 5: e11014.

5. Potthoff MJ, Olson EN (2007) MEF2: a central regulator of diverse developmental programs. Development 134: 4131-4140.

6. Saegusa M, Hashimura M, Kuwata T (2012) Sox4 functions as a positive regulator of beta-catenin signaling through upregulation of TCF4 during morular differentiation of endometrial carcinomas. Lab Invest 92: 511-521.

7. Wan X, Ji W, Mei X, Zhou J, Liu JX, et al. (2010) Negative feedback regulation of Wnt4 signaling by EAF1 and EAF2/U19. PLoS One 5: e9118.

8. Bernard P, Fleming A, Lacombe A, Harley VR, Vilain E (2008) Wnt4 inhibits beta-catenin/TCF signalling by redirecting beta-catenin to the cell membrane. Biol Cell 100: 167-177.

9. Park TJ, Haigo SL, Wallingford JB (2006) Ciliogenesis defects in embryos lacking inturned or fuzzy function are associated with failure of planar cell polarity and Hedgehog signaling. Nat Genet 38: 303-311.

10. Kim SK, Shindo A, Park TJ, Oh EC, Ghosh S, et al. (2010) Planar cell polarity acts through septins to control collective cell movement and ciliogenesis. Science 329: 1337-1340.

11. Pyati UJ, Cooper MS, Davidson AJ, Nechiporuk A, Kimelman D (2006) Sustained Bmp signaling is essential for cloaca development in zebrafish. Development 133: 2275-2284.
